# Reference Gene Selection for qPCR Is Dependent on Cell Type Rather than Treatment in Colonic and Vaginal Human Epithelial Cell Lines

**DOI:** 10.1371/journal.pone.0115592

**Published:** 2014-12-19

**Authors:** Annette V. Jacobsen, Bisrat T. Yemaneab, Jana Jass, Nikolai Scherbak

**Affiliations:** 1 School of Science and Technology, Örebro University, Örebro, Sweden; 2 School of Biomedical Sciences, Charles Sturt University, Wagga Wagga, Australia; Taipei Medical University, Taiwan

## Abstract

The ability of commensal bacteria to influence gene expression in host cells under the influence of pathogenic bacteria has previously been demonstrated, however the extent of this interaction is important for understanding how bacteria can be used as probiotics. Real-time quantitative polymerase chain reaction is the most sensitive tool for evaluating relative changes to gene expression levels. However as a result of its sensitivity an appropriate method of normalisation should be used to account for any variation incurred in preparatory experimental procedures. These variations may result from differences in the amount of starting material, quality of extracted RNA, or in the efficiency of the reverse transcriptase or polymerase enzymes. Selection of an endogenous control gene is the preferred method of normalisation, and ideally a proper validation of the gene's appropriateness for the study in question should be performed. In this study we used quantitative polymerase chain reaction data and applied four different algorithms (geNorm, BestKeeper, NormFinder, and comparative ΔC_q_) to evaluate eleven different genes as to their suitability as endogenous controls for use in studies involving colonic (HT-29) and vaginal (VK2/E6E7) human mucosal epithelial cells treated with probiotic and pathogenic bacteria. We found phosphoglycerate kinase 1 to be most appropriate for HT-29 cells, and ribosomal protein large P0 to be the best choice for VK2/E6E7 cells. We also showed that use of less stable reference genes can lead to less accurate quantification of expression levels of gene of interest (GOI) and also can result in decreased statistical significance for GOI expression levels when compared to control. Additionally, we found the cell type being analysed had greater influence on reference gene selection than the treatment performed. This study provides recommendations for stable endogenous control genes for use in further studies involving colonic and vaginal cell lines after bacterial challenge.

## Introduction

Mucosal epithelial cells have an important role in monitoring and stimulating the innate and adaptive immune responses to a variety of antigens and pathogens [1]. The influence of commensal bacteria in modulation of these responses is of particular interest considering their therapeutic potential as probiotics. Probiotic bacteria are defined as live organisms that confer a health benefit to the host when applied in sufficient amount, and include lactic acid bacteria found in the gastrointestinal and genitourinary tracts [2]. These have been shown to interact with host microbiota and protect against pathogen colonisation using strategies such as competitive adhesion, production of inhibitory or bactericidal substances, and displacement of biofilms [3]–[5]. In addition to this, it has also been demonstrated that certain lactobacilli can influence immune response by modulating gene expression of host cells [6]–[8]. Disruption of microbial balance in the colon and vagina has been associated with a number of pathologies, including irritable bowel disease, colon cancer, and candidiasis of the vagina, and the use of probiotic species in combatting these disruptions is a promising field of research [7], [9].

The extent to which commensal bacteria affect gene regulation in host cells is important in understanding their impact on immune modulation at a molecular level, and may provide insight into how they can be utilised as probiotics. Currently the real-time quantitative polymerase chain reaction (qPCR) is accepted as the standard for assessing changes to gene expression [10]. However, as a result of its sensitivity, real-time qPCR is easily affected by small errors in the experimental process, which are then amplified by the nature of the reaction [11]. Experimental variation may include differences in RNA extraction efficiencies, differences in reverse transcriptase (RT) and PCR enzyme efficiencies, and differences in quality and quantity of starting material [12], [13]. This experimental variation has the potential to mask the true biological variation if it is not appropriately accounted for. To correct for this variation a number of strategies have been proposed, including normalising against total RNA, genomic DNA, or incorporation of an introduced RNA molecule [13]. Although the latter method, particularly, shows promise, the generally accepted method of normalisation for qPCR involves the use of endogenous control genes [12]. These control genes, also known as reference or housekeeping genes, should be stably expressed in all cells or tissues under investigation, and should not be differentially regulated by the experimental process [14]. Reference genes can limit errors resulting from experimental variation as they are subject to the same experimental procedures as the target genes [11].

Selection of a good reference gene can greatly improve reliability, although the use of a reference gene that has not yet been validated may result in erroneous conclusions [14]. Despite this, many studies still use traditional reference genes such as glyceraldehyde-3-phosphate dehydrogenase (*GAPDH*) and actin, beta (*ACTB*) without determining their suitability [15], [16]. Traditionally, housekeeping genes, responsible for essential intracellular processes, were used as references since they are expressed at reliable levels in all cells and are easy to detect. However it is now recognised that their expression can be affected by experimental conditions, therefore references should be validated for a particular set of conditions prior to use [12], [15], [17].

To assist in determining the most appropriate gene for each individual circumstance, a number of different validation strategies have been developed. This includes the comparative ΔC_q_ method, which measures the changes in quantification cycle (C_q_) of both treated and untreated samples, and algorithms such as geNorm, NormFinder and BestKeeper, which suggest the use of multiple internal control genes [14], [18], [19]. geNorm uses a geometric mean of relative expression levels to determine the most stable reference gene, and then applies pairwise variation to determine the number of candidates required. Normfinder uses a model-based approach based on both intra- and inter-group variation between different treatment groups. BestKeeper calculates a geometric mean based on raw C_q_ values, and then applies pairwise correlation and regression analyses to determine the most stable combination. The increasing trend to use more than one reference gene is based on the assumption that it is unlikely that any single gene is totally free of regulation in all circumstances, so the use of multiple internal controls is likely to give more reliable results [11], [20].

We assessed a number of candidate reference genes for suitability to normalise a study involving treatment of colonic (HT-29) and vaginal (VK2/E6E7) human mucosal cell lines after treatment with different probiotic strains and pathogenic bacteria. We found that suitable reference genes were cell line specific rather than treatment specific, with phosphoglycerate kinase 1 (*PGK1*) being the most stable candidate for normalisation of colonic cells, and ribosomal protein large P0 (*RPLP0*) having the lowest variation for vaginal cells. In addition to this, we found normalisation may be improved by the use of multiple internal control genes, particularly when increased sensitivity is required. Finally, this study provides validation of reference genes for future use in gene expression studies involving this model, which could be useful in understanding how these interactions influence the immune response at a molecular level.

## Methods

### Candidate reference gene selection

The eleven genes selected as reference candidates and their primary functions are shown in Table 1. Candidate genes were selected taking into account a range of primary functions so to limit potential for co-regulation affecting the results. Five of the selected genes were commonly used reference genes (*GAPDH*, *ACTB*, *PGK1*, *PPIA*, and *RPLP0*), two were selected based on literature recommendations (*POLR2A* and *DEFB1*), two were selected based on observation of relatively stable expression in a primary screen (*DICER1* and *DROSHA*) and the final two candidate genes (*TMEM222* and *MVK*) were selected using RefGenes (http://www.refgenes.org/rg/), a software program that suggests candidate reference genes based on microarray analysis of similar tissue types under a variety of experimental conditions [11], [21], [22].

**Table 1:** Primer sequences used for qPCR analysis.

Gene	Accession No.	Forward (F) and Reverse (R) Primers	Exon F, R	Gene Function
POLR2A	NM_000937	F:AAGTTCAACCAAGCCATTGCG R:GACACACCAGCATAGTGGAAGG	19–20, 20	mRNA synthesis
TMEM222	NM_032125	F:TCTACGGGAAGTACGTCAGC R:CCATCACCGGAGGTTAAAGACC	2–3, 3	membrane integrity
MVK	NM_000431	F:AAGGTAGCACTGGCTGTATCC R:CCAATGTTGGGTAAGCTGAGG	6–8, 8	cholesterol synthesis
DEFB1	NM_005218	F:GTTCCTGAAATCCTGGGTGTTG R:CTGTGAGAAAGTTACCACCTGAG	1, 1–2	immune response
PPIA	NM_021130	F:GCTTGCTGGCAGTTAGATGTC R:AGAGGTCTGTTAAGGTGGGC	5, 5	protein folding
GAPDH	NM_002046	F:ATTTGGCTACAGCAACAGGG R:TCAAGGGGTCTACATGGCA	10, 10	glycolytic enzyme
RPLP0	NM_001002	F:ACAATGGCAGCATCTACA R:GTAATCCGTCTCCACAGA	6, 7	protein synthesis
PGK1	NM_000291	F:GAGATGATTATTGGTGGTGGAA R:AGTCAACAGGCAAGGTAATC	7, 8	glycolytic enzyme
DICER1	NM_177438	F:GTACGACTACCACAAGTACTTC R:ATAGTACACCTGCCAGACTGT	24, 25	small RNA synthesis
DROSHA	NM_013235	F:GCAGCGCAAAGGCAAGACGC R:AGGCGGGGAGACTGTGATCCG	10, 11	small RNA synthesis
ACTB	NM_001101.3	F:CACACAGGGGAGGTGATAGC R:GACCAAAAGCCTTCATACATCTCA	6, 6	cellular structure

POLR2A - polymerase (RNA) II (DNA directed) polypeptide A; TMEM222 - transmembrane protein 222; MVK - mevalonate kinase; DEFB1 - defensin beta 1; PPIA - peptidylprolyl isomerase; GAPDH - glyceraldehyde-3-phosphate dehydrogenase; RPLP0 - ribosomal protein, large, P0; PGK1 - phosphoglycerate kinase 1; DICER1 - dicer 1, ribonuclease type III; DROSHA - drosha, ribonuclease type III; ACTB – actin, beta.

### Primer design and validation

A list of primers used in this study is shown in Table 1. Sequences for primers not designed in this study (*RPLP0* and *PGK*) were supplied by Sigma-Aldrich (St Louis, MO, USA), and the remaining primers were designed using either QuantPrime (http://www.quantprime.de) or NCBI/Primer-BLAST (http://www.ncbi.nlm.nih.gov/tools/primer-blast/)[23], [24]. All primers were assessed for specificity against a human mRNA database using Primer-BLAST, and potential for primer-dimer associations and secondary structure formation was assessed using Beacon Designer (http://www.premierbiosoft.com/qpcr/index.html) and UNAFold (http://eu.idtdna.com/UNAFold) software. Primer oligonucleotides were built by Eurofins MWG Operon (Luxembourg, Luxembourg).

Primers were determined to be effective via conventional PCR and product and size was confirmed by electrophoresis on 1.5% agarose gels. Linear range, approximate efficiency and specificity of primers were assessed with qPCR and melting curve analysis using eight 10 fold serial dilutions of PCR product starting with a 1∶10000 dilution.

### Bacterial strains and culture conditions

*Lactobacillus acidophilus* NCFM was isolated from Ultra Dophilus DF dietary supplement powder (Metagenics, Aliso Viejo, CA, USA) and *Lactobacillus rhamnosus* GR-1 was kindly donated by Gregor Reid. Bacteria were grown on de Man Rogosa Sharp (MRS) agar (Becton Dickinson [BD], Franklin Lakes, NJ, USA) in GasPak EZ Anaerobe Generating Pouch Systems (BD, Franklin Lakes, NJ, USA), at 37°C for 24 hours. One colony was inoculated in a tube filled to the top with MRS liquid media (BD, Franklin Lakes, NJ, USA) and kept vertical, with no shaking, for 24 hours at 37°C to create anaerobic conditions. One mL of the culture was centrifuged at 3000×*g* for 10 minutes. The pellet was washed twice with sterile phosphate buffer saline (PBS; pH 7.4) and finally resuspended in 1 mL sterile PBS. Bacteria were quantified by plating serial dilutions of the culture onto MRS agar, incubating anaerobically at 37°C overnight, and counting of colony forming units (CFUs).

To prepare heat killed (HK) *Escherichia coli* GR-12 (ATCC, Manassas, VA, USA), bacteria were grown in Luria Bertani agar (LB; Sigma-Aldrich, St Louis, MO, USA) at 37°C overnight. One colony was inoculated in 5 mL of LB broth (Sigma-Aldrich, St-Louis, MO, USA) and incubated at 37°C overnight with shaking at 200 r.p.m. The overnight cell culture was centrifuged at 3000×g for 10 minutes. The pellet was washed twice with sterile PBS (pH 7.4), resuspended in 500 µL sterile PBS and heated at 70°C for 1 hour in a water bath. To confirm loss of viability, the heat-treated bacterial cells were plated on LB agar overnight. In parallel with heat killing, bacteria were quantified by plating serial dilutions of the culture onto LB agar, incubating at 37°C overnight, and counting of CFUs. Stock cultures were maintained at −20°C in 10% glycerol.

### Epithelial cell culture

For this study HT-29 human colonic epithelial cells (HTB38; ATCC, Manassas, VA, USA) and VK2/E6E7 human vaginal epithelial cells (CRL2616; ATCC, Manassas, VA, USA) were selected as representative mucosal cell lines. HT-29 cells were cultured in McCoy's 5A media (Hyclone, Waltham, MA, USA) supplemented with 10% foetal bovine serum (FBS; Hyclone, Waltham, MA, USA). VK2/E6E7 cells were cultured in Keratinocyte-Serum Free medium supplemented with bovine pituitary extract and human recombinant endothelial growth factor 1–53 (all from Life Technologies, Stockholm, Sweden), with additional calcium chloride to obtain a final concentration of 0.4 mM. Subculturing was performed at approximately 70% confluence for both cell lines using a 0.25% trypsin, 0.53 mM EDTA solution. All active cell cultures were maintained at 37°C in 5% CO_2_. Stock cultures were frozen in HT-29 growth medium with 5% DMSO, and stored in liquid nitrogen.

### Cell treatment

Epithelial cells were seeded in 6 well plates (BD, Franklin Lakes, NJ, USA) at a density of 3×10^5^ cells/well and were grown to 90% confluence in the appropriate growth media, to achieve a cell density approximately 3×10^6^ cells/well. On the day of treatment, the media was replaced with fresh media and allowed to acclimatise for one hour. The cells were then treated with either HK *E. coli* GR-12 (equivalent to 3×10^7^ CFU/mL), *L. rhamnosus* GR-1 (10^8^ CFU/mL), *L. acidophilus* NCFM (10^8^ CFU/mL), or with a combination of HK *E. coli* GR-12 and one of the *Lactobacillus* strains at the concentrations previously stated. The treated cells were incubated at 37°C in 5% CO_2_ for 3 hours. The cells were harvested for total RNA extractions using the methods detailed below. All experiments were performed in triplicate (n = 3).

### RNA extraction and cDNA synthesis

Total RNA was extracted using the Nucleospin II kit (Macherey-Nagel, Düren, Germany). RNA concentration and purity was estimated using a NanoVue Spectrophotometer (GE Healthcare, Little Chalfont, UK), and assessed for quality using agarose gel electrophoresis. cDNA was synthesised in 20 µL reactions using a qScript kit (Quanta Bioscience, Gaithersburg, MD, USA) from 1 µg of the extracted RNA as per the manufacturer's instructions. Synthesised cDNA was then diluted ten times for use as a template for the qPCR reaction.

### Conventional polymerase chain reaction

Conventional PCR was performed using a Hybaid PCR Sprint thermocycler (Thermo Scientific, Waltham, MA, USA). Twenty five µL reactions were prepared using Maxima Hot Start *Taq* Buffer (10X; Thermo Scientific, Waltham, MA, USA), with 2.5 mM MgCl_2_, 0.3 µM of each forward and reverse primers (except *ACTB)*, 0.2 mM dNTP, 1 U Maxima Hot Start *Taq* DNA Polymerase, and 25 ng template cDNA. In the case of *ACTB*, primer concentrations needed to be optimised and were used at 0.2 µM of forward primer and 0.07 µM of reverse primer. The amplification consisted of a 4 minute initial denaturation at 95°C, then 40 cycles of denaturation for 30 seconds at 95°C, annealing for 30 seconds at 60°C, and extension for 30 seconds at 72°C. The PCR was concluded with a final 5 minute extension at 72°C. Confirmation of a product of the appropriate size was by electrophoresis on 1.5% agarose gels.

### Quantitative polymerase chain reaction

The qPCR reaction was performed with the ABI Prism 7900HT Sequence Detection System (Applied Biosystems, Foster City, CA, USA) in clear 96 well fast plates. Fifteen µL reactions were prepared with the Maxima SYBR Green qPCR Master Mix (x2) (Thermo Scientific, Waltham, MA, USA), using a 0.2 µM end concentration of each of the forward and reverse primers (except *ACTB*, in which primer concentrations were as above), 267 nM Rox, and 25 ng template cDNA. Amplification was carried out with a preliminary 2 minute UDG pretreatment at 50°C followed by a 10 minute initial denaturation at 95°C, followed by 40 cycles of denaturation for 15 seconds at 95°C and annealing/extension for 60 seconds at 60°C. All qPCR amplifications were concluded with an additional dissociation step for melting curve analysis, and no-template and no reverse transcriptase controls were included for each experiment.

### Reference gene data analysis

All qPCR data was recorded in relation to quantification cycle (C_q_), also known as cycle threshold (C_t_), as recommended by the MIQE guidelines [16]. qPCR was performed on six treatment groups for each cell line, inclusive of a control group, with three biological replicates, as described under cell treatment. These were pooled together for the purpose of reference gene analysis, giving a total of eighteen samples to represent each cell line. For analysis purposes, primer efficiency and C_q_ values of the candidate reference genes were generated with Real-time PCR Miner (http://www.miner.ewindup.info/; Version 4.0), using the raw fluorescence data generated during the qPCR reactions [25]. Assessment of candidate gene performance was determined using four different algorithms: geNorm, BestKeeper, NormFinder and the comparative ΔC_q_ method [18], [19], [26], [27]. Corrections for efficiency were performed for all four algorithms using the formula C_q_(adj)  =  [log(1+E)/log(2)] × C_q_(exp), where E = % efficiency/100, C_q_(exp)  =  experimental C_q_, and C_q_(adj)  =  adjusted C_q_.

All candidate reference genes were assessed for stability using the geNorm algorithm contained in the qbasePLUS software program (http://www.biogazelle.com/; Version 2.4) [27]. C_q_ values were entered into qBasePLUS and were converted to relative quantities, which were then used for further calculations. The geNorm algorithm determines the most stable combination of reference target based on the geometric mean of the most stable control genes to generate a stability (M) value. The number of reference genes required for accurate normalization is then determined by calculating the pairwise variation (V_n/n+1_).

The NormFinder Excel applet also bases its calculations on relative expression levels and was able to assess reference gene stability based on both intra- and inter-group variation, where groups represented different experimental conditions (http://moma.dk/normfinder-software). NormFinder analysis then determines the most stable reference genes using a model-based approach, which takes into account both of these variations.

Contrary to geNorm and NormFinder, BestKeeper input is raw C_q_ values (http://www.gene-quantification.com/bestkeeper.html). From this, a geometric mean of candidate reference targets is calculated and used as a basis of comparison to determine the ideal reference targets using pairwise correlation analysis. Individual gene stability is also assessed, and is expressed as a standard deviation (s.d.).

The final method used for analysis was the comparative ΔC_q_ method, which determines the stability of reference candidates based on the average s.d. of the change in C_q_ values of a particular gene when compared to all other genes being assessed, with the lowest s.d. considered to signify the highest stability.

Overall ranking of reference gene candidates was determined using the geometric mean of the rankings generated from the individual algorithms, as previously described [28].

### Simulation of analysis of gene expression

Expression values used for the reference genes in this study were those generated from the qPCR analysis and were adjusted for efficiency as described in a previous section. Expression values of the hypothetical gene of interest (hypGOI) were generated to show both increases and decreases of two-fold and five-fold when normalised against the most stable reference genes determined for both cell lines. Expression values for the hypGOI were also generated so that their variation produced a standard deviation corresponding to 0.2 of the average expression when normalised to the best-ranked gene. The effect of using a lower ranked reference gene was assessed by renormalisation of the hypothetical gene of interest against one middle-ranked and two poorly ranked reference genes for each cell line. Statistical significance was assessed using a Student's t-test.

## Results

### Primer efficiency and specificity

Primers were assessed for efficiency, linear dynamic range and specificity using qPCR after conducting a series of ten-fold dilutions from 1∶10^4^ to 1∶10^11^. Efficiency (E) was calculated using the formula E  =  (10^(−1/slope)^-1) ×100. The linear range of primers was between C_q_ 9 –34, and, using linear regression, all primers were found to have approximate efficiencies between 89–104%, with r^2^>0.99. The efficiencies of all primers were within the guidelines recommended by Thermo Scientific (90–105%), with the exception of ACTB (89%). However, as the linear dynamic range for this primer was good (C_q_ 10–32), and the gene was strongly expressed in both cell lines (C_q_∼12–14), it was included in the analysis. Results are summarised in Table 2.

**Table 2:** Primer efficiencies and linear dynamic range.

Primer	Linear range (C_q_)	r^2^	Efficiency
GAPDH	9 to 27	0.9997	98%
PGK1	11 to 29	0.9981	91%
PPIA	10 to 27	0.9998	104%
RPLP0	11 to 30	0.9992	93%
TMEM222	10 to 36	0.9998	93%
POLR2A	11 to 29	0.9998	98%
MVK	10 to 28	0.9997	96%
DEFB1	10 to 32	0.9998	99%
ACTB	10 to 32	0.9995	89%
DICER1	9 to 32	0.9994	100%
DROSHA	10 to 34	0.9983	99%

### Real-time qPCR validation

**Figure 1:**
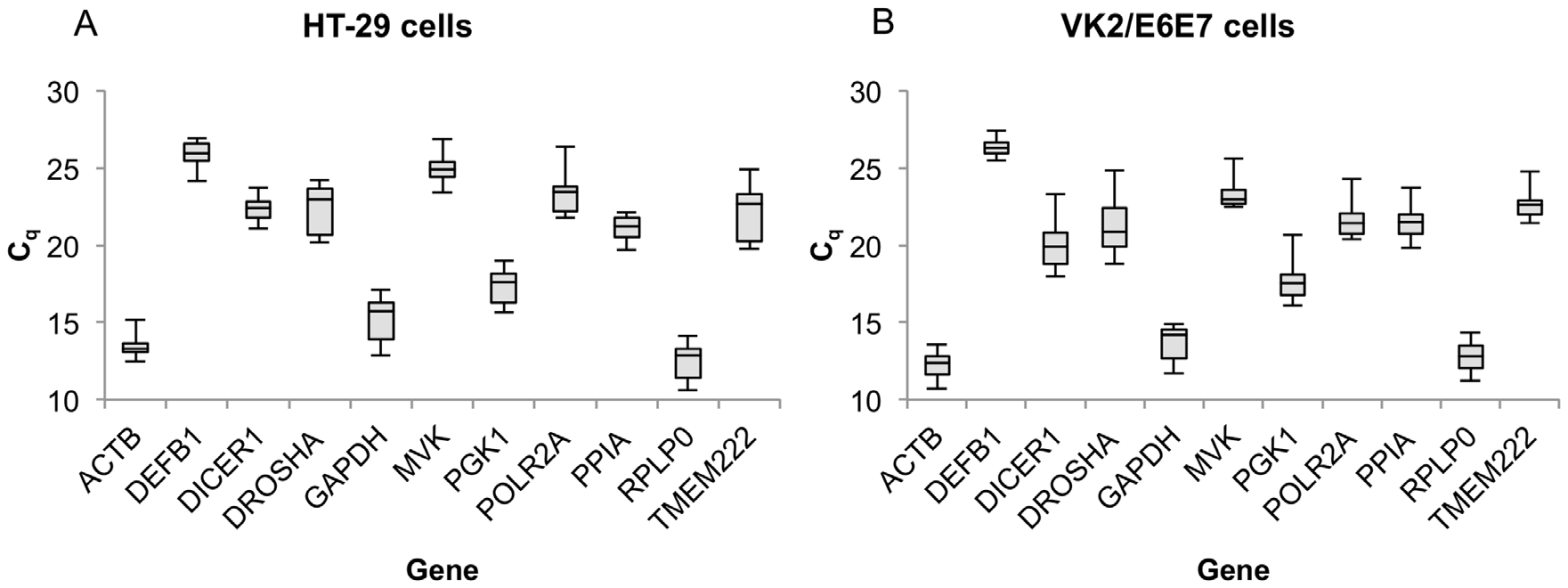
Range of C_q_ values for the colonic HT-29 (A) and vaginal VK2/E6E7 (B) cell lines. The range of C_q_ values as measured by qPCR for both the HT29 (A) and VK2/E6E7 (B) cells. The plots highlight the median (centre line), maximum and minimum (whiskers), and 1^st^ and 3^rd^ quartile marks (boxes) of the data. All C_q_ values measures were within the bounds of the linear dynamic range as determined by standard curve analysis.

C_q_ values generated from the qPCR reactions were within the linear dynamic range of their respective primers as determined by the dilution series. Negative controls were classed as successful where the ΔC_q_ between the lowest negative control and the highest template well was greater than 7, and this condition was met in all assays. A summary of the range of generated C_q_ values for all genes is shown in Fig. 1.

### geNorm analysis

**Figure 2:**
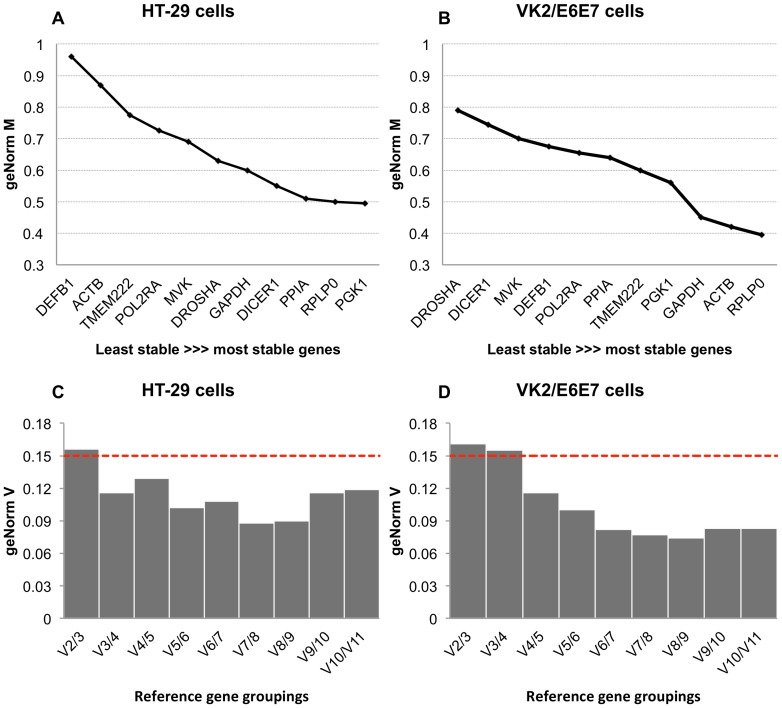
geNorm analysis of colonic HT-29 (A and C) and vaginal VK2/E6E7 (B and D) cell lines. The geNorm stability (M) values are shown in order of increasing stability for both colonic HT-29 (A) and vaginal VK2/E6E7 cell lines (B). The steeper the line between two adjacent genes, the greater the impact removal of this gene has on overall reference gene stability. The geNorm variation (V) assessment is also summarised for colonic HT-29 (C) and vaginal VK2/E6E7 (D) cell lines. A lower value in this analysis indicates less variation and increased accuracy of normalisation. The cut off at which addition of further reference genes is not presumed to improve normalisation ability is 0.15, and is shown by the dashed red line.

For the colonic cell line HT-29, the most stable individual gene was found to be *PGK1* (M = 0.727, CV = 0.196), followed by *DICER1*, *PPIA*, *RPLP0*, *GAPDH*, *MVK*, *DROSHA*, *POLR2A*, *TMEM222*, *ACTB* and *DEFB1*, in order of decreasing stability. All genes except *TMEM222*, *ACTB* and *DEFB1* showed high expression stabilities for heterogeneous samples (M<1). By elimination of the least stable genes, the best combination of reference genes assessed by genorm^PLUS^ for this sample set was *PGK1*, *PPIA* and *RPLP0* (M = 0.510, V = 0.117; Fig. 2A, C).

For the vaginal cell line VK2/E6E7, the most stable individual gene was found to be *RPLP0* (M = 0.643, CV = 0.210), followed by *TMEM222*, *PPIA*, *POLR2A*, *PGK1*, *ACTB*, *GAPDH*, *DEFB1*, *DICER1*, *MVK*, and *DROSHA*, in order of decreasing stability. All samples showed high expression stabilities (M<1), making them suitable reference candidates. For these cells, geNorm analysis indicated that use of four reference genes would give the best stability (Fig. 2B, D), and suggested the use of *RPLP0*, *ACTB*, *GAPDH*, and *PGK1* (M = 0.555, V = 0.116).

### NormFinder analysis

**Figure 3:**
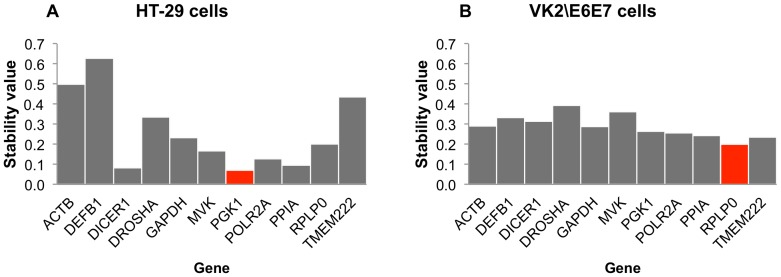
NormFinder analysis of colonic HT-29 (A) and vaginal VK2/E6E7 (B) cell lines. NormFinder calculated stability values based on relative expression values. A lower value indicates superior stability. Best ranking candidates for each gene are indicated in red.

NormFinder results for the colonic cell line also determined *PGK1* to be the most stable reference target (stability value  = 0.070), followed by *DICER, PPIA, POLR2A, MVK, RPLP0, GAPDH, DROSHA, TMEM222, ACTB,* and *DEFB1* (Fig. 3A). However, the result for an ideal combination was quite different, being assessed as *PGK1* and *DICER1* (combined stability value  = 0.057).

For the vaginal cell line, NormFinder also found *RPLP0* to be the most stable individual candidate (stability value 0.200), followed by *TMEM222, PPIA, POLR2A, PGK1, GAPDH, ACTB, DICER, DEFB1, MVK,* and *DROSHA* (Fig. 3B). Assessment of the most stable pair of reference targets indicated an advantage to using the combination of *PGK1* and *PPIA* (combined stability value  = 0.132).

### BestKeeper analysis

Unlike the other two methods, BestKeeper requires the user to actively participate in eliminating less suitable candidates to arrive at the ideal reference gene combination. In the colonic cell line, *DEFB1* and *ACTB* were first eliminated, having little correlation to the BestKeeper ideal for this data set. Then *TMEM222*, *POLR2A,* and *MVK* were all excluded from further analysis due to having a s.d. >1 C_q_. From this group of six final candidates, genes were eliminated based on their coefficient of correlation [r] to the BestKeeper index, arriving at a final data set of three genes: *PGK1*, *DICER*, and *POLR2A*. BestKeeper does not recommend the use of less than three genes for normalisation of a qPCR study, however from this data set the gene that correlated most closely to the BestKeeper index was *PGK1* ([r]  = 0.98; p = 0.001).

In the vaginal cell line overall correlation between the candidate genes was much better. *DROSHA* and *DICER1* were eliminated first due to a s.d. >1 C_q_, with MVK eliminated next, having a relatively low [r] value (<0.7). Further eliminations were performed based on [r] value to arrive at a final set of three genes all with [r] values >0.95: *RPLP0*, *ACTB*, and *GAPDH*. Of these, *RPLP0* was found to correlate most closely with the BestKeeper index ([r]  = 0.98; p = 0.001). A summary of some key BestKeeper statistics for the top six genes in each data set is found in Table 3.

**Table 3:** Summary of key statistics after BestKeeper analysis.

Colonic HT-29 cells						
	DICER1	MVK	PGK1	POLR2A	PPIA	RPLP0
s.d. [±Cq]	0.68	0.71	0.95	0.97	0.67	0.88
coeff. of corr. [r]	0.947	0.823	0.988	0.931	0.935	0.877
p-value	0.001	0.001	0.001	0.001	0.001	0.001
Power [x-fold]	1.77	1.79	2.23	2.30	1.72	1.97

Vaginal VK2/E6E7 cells						
	ACTB	GAPDH	PGK1	POLR2A	PPIA	RPLP0
s.d. [±Cq]	0.70	0.99	0.82	0.85	0.80	0.77
coeff. of corr. [r]	0.921	0.938	0.903	0.851	0.898	0.982
p-value	0.001	0.001	0.001	0.001	0.001	0.001
Power [x-fold]	1.75	2.17	2.04	1.97	2.01	1.95

The standard deviation (s.d.) corresponds to the individual gene stability and the coefficient of correlation, with its respective p-value, corresponds to how closely the candidate reference gene resembles the BestKeeper ideal normalisation factor after repeated pair-wise analysis. The power value is determined by regression analysis, using fold change (x-fold) as a reference point, and a smaller value indicates a better reference candidate. Values for genes eliminated in earlier stages of analysis are not shown.

### Comparative ΔC_q_ analysis

As a final method of comparison, the comparative ΔC_q_ method was used to generate a ranking for both samples and was found to compare favourably with the other methods used. For the colonic cell line the top three and bottom four genes were identical to those determined using the NormFinder method, and in the vaginal cell line the top five and bottom two genes were also identical to the NormFinder evaluation, with only minor changes to the other positions. A summary of the full results can be seen in Table 4.

**Table 4:** Mean standard deviation (s.d.) of reference genes using the comparative ΔC_q_ method.

Colonic HT-29 cells		Vaginal VK2/E6E7 cells	
Gene	Mean s.d.	Gene	Mean s.d.
PGK1	0.705	RPLP0	0.643
DICER1	0.736	TMEM222	0.698
PPIA	0.758	PPIA	0.721
GAPDH	0.808	POL2R2A	0.733
POLR2A	0.820	PGK	0.738
RPLP0	0.869	ACTB	0.758
MVK	0.885	GAPDH	0.777
DROSHA	0.916	DEFB1	0.859
TMEM222	1.117	DICER1	0.866
ACTB	1.259	MVK	0.886
DEFB1	1.439	DROSHA	0.997

### Overall reference gene ranking

To determine the best overall reference candidates we determined the geometric mean of the individual rankings assessed by each of the four algorithms. We found the reference candidates in order of stability for the colonic cell line HT-29 were *PGK1*, *DICER*, *PPIA, RPLP0, POLR2A, GAPDH*, *DROSHA*, *MVK, TMEM222*, *ACTB* and *DEFB1*, and for the vaginal cell line VK2/E6E7 were *RPLP0, TMEM222*, *ACTB*, *PPIA*, *GAPDH*, *PGK1*, *POLR2A, DEFB1*, *DICER1*, *MVK* and *DROSHA* (Table 5).

**Table 5:** Comprehensive ranking of reference gene candidates by calculation of a geometric mean.

Colonic HT-29 cells					
geNorm	BestKeeper	NormFinder	ΔC_q_	Ranking	Mean
PGK1	PGK1	PGK1	PGK1	**PGK1**	1.000
RPLP0	DICER1	DICER1	DICER1	**DICER1**	2.378
PPIA	POLR2A	PPIA	PPIA	**PPIA**	3.224
DICER1	PPIA	POLR2A	GAPDH	**RPLP0**	4.356
GAPDH	RPLP0	MVK	POLR2A	**POLR2A**	4.681
DROSHA	MVK	RPLP0	RPLP0	**GAPDH**	5.595
MVK	GAPDH	GAPDH	MVK	**DROSHA**	6.055
POLR2A	DROSHA	DROSHA	DROSHA	**MVK**	6.192
TMEM222	TMEM222	TMEM222	TMEM222	**TMEM222**	9.000
ACTB	ACTB	ACTB	ACTB	**ACTB**	10.000
DEFB1	DEFB1	DEFB1	DEFB1	**DEFB1**	11.000

Vaginal VK2-E6/E7 cells					
geNorm	BestKeeper	NormFinder	ΔC_q_	Ranking	Mean
RPLP0	RPLP0	RPLP0	RPLP0	**RPLP0**	1.000
ACTB	ACTB	TMEM222	TMEM222	**TMEM222**	3.440
GAPDH	GAPDH	PPIA	PPIA	**ACTB**	3.600
PGK1	PPIA	POL2RA	POLR2A	**PPIA**	3.834
TMEM222	PGK1	PGK1	PGK1	**GAPDH**	4.409
PPIA	POLR2A	GAPDH	ACTB	**PGK1**	4.729
POLR2A	TMEM222	ACTB	GAPDH	**POLR2A**	5.091
DEFB1	DEFB1	DICER1	DEFB1	**DEFB1**	8.239
MVK	MVK	DEFB1	DICER1	**DICER1**	9.212
DICER1	DICER1	MVK	MVK	**MVK**	9.487
DROSHA	DROSHA	DROSHA	DROSHA	**DROSHA**	11.000

### Analysis of influence of bacterial species on reference gene selection

**Figure 4:**
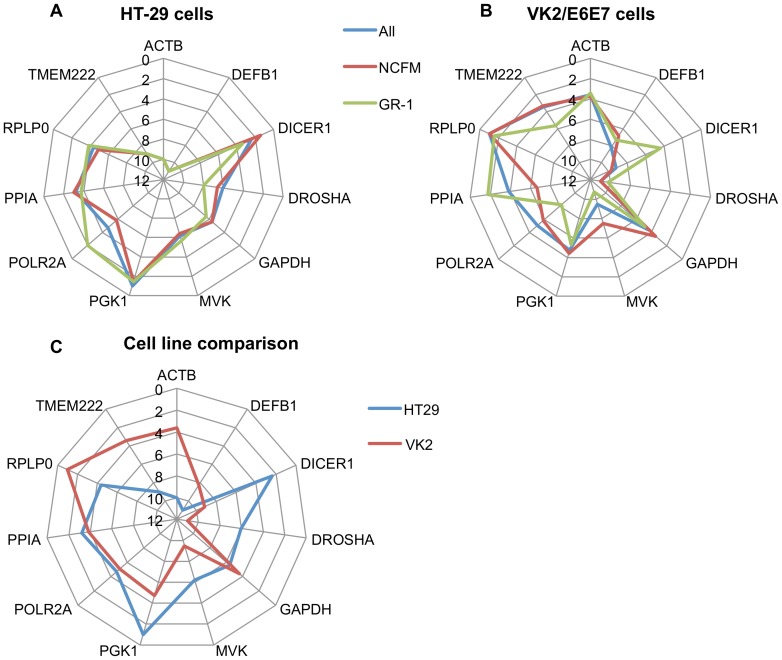
Comparison of reference gene rankings based on cell type and bacterial challenge. Gene names are around the outside of the wheel and follow the radial lines. The numbered concentric lines indicate the geometric ranking. The further away from the centre the line is, the more stable the reference gene. Each data set is plotted with a different colour. The degree to which the coloured lines overlap indicate how well the data sets correlate to each other. **A.** Colonic HT-29 cells. Blue: combined data set. Red: *Lactobacillus acidophilus* NCFM used as the commensal strain. Green: *Lactobacillus rhamnosus* GR-1 used as the commensal strain. **B.** Vaginal VK2/E6E7 cells. Blue: combined data set. Red: *L. acidophilus* NCFM used as the commensal strain. Green: *L. rhamnosus* GR-1 used as the commensal strain. **C.** Comparison of combined data sets of the HT-29 and VK2/E6E7 cell lines. Blue: HT-29 cells. Red: VK2/E6E7 cells.

To further determine reference gene stability we subdivided our data set to determine if there was any difference in reference stability dependent on the species of *Lactobacillus* we were using. A summary of data from each of the algorithms used and the calculated geometric mean can be found in S1–S3 Figs. We then compared geometric means the overall data sets for each cell line with the subdivided data sets of *L. acidophilus* NCFM and *L. rhamnosus* GR-1, using radar graphs to illustrate the differences in ranking (Figs. 4A,B). This graphing method highlights the differences in reference gene suitability as a result of the Lactobacillus species used for treatment in both cell lines. In the colonic cell line these differences are small, with an average difference of 1.04 ranking points across the experimental models, and only *POLR2A* showing a difference of greater than two ranking points. The vaginal cell line showed slightly greater variability, with an average difference of 2.21 ranking points across models. Only four genes had differences of greater than two ranking points (*PPIA*, *DICER1*, *MVK*, and *POLR2A*), with both *PPIA* and *DICER* showing differences in rankings by approximately five points. None of these four genes were in the top four places in the overall ranking.

Radar graphs were used to illustrate the differences between the comprehensive rankings of both cell lines (Fig. 4C). A clear difference between the cell lines was assessed, with an average difference of 3.55 ranking points. Overall, eight genes showed ranking differences of greater than two points, including three out of the top four genes for both cell lines. The largest differences involve *TMEM222* and *ACTB* which ranked 2^nd^ and 3^rd^ in the vaginal cell line being ranked 9^th^ and 10^th^ in the colonic samples, and *DICER1* which ranked 2^nd^ in the colonic cell line placing 9^th^ in the vaginal cells.

### Impact of reference gene selection on gene expression studies

To determine the impact of using a poorly ranked reference gene on an expression study, we performed a hypGOI expression analysis using data from both HT29 and VK2/E6E7 cells treated with *L. acidophilus* NCFM as a test case. Simulation of expression of hypGOI in HT29 was assessed using highly ranked *PGK1*, and three lesser ranked genes: *MVK* (middle-ranked), *ACTB* and *DEFB1* (both are poorly ranked). For the VK2/E6E7 cells, we used the highly ranked *RPLP0* along with *PGK1* (middle-ranked), *DICER1* and *MVK* (both are poorly ranked).

**Figure 5:**
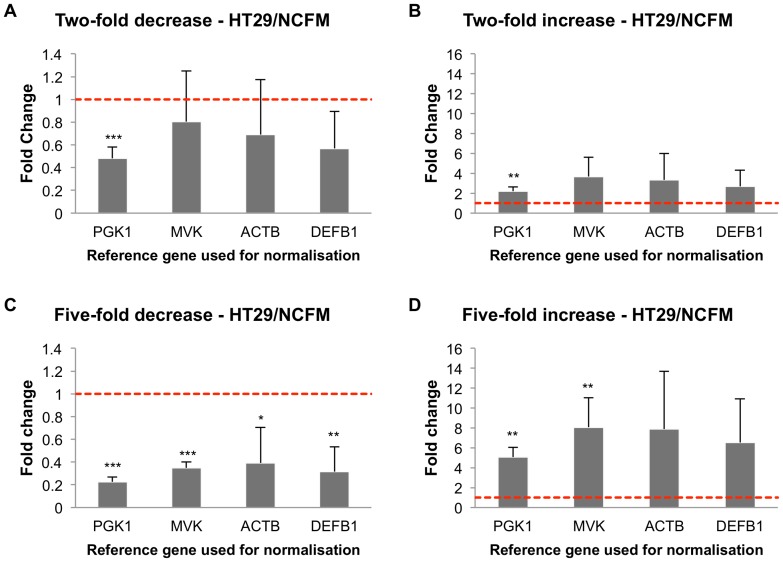
Simulation of hypGOI expression study in HT-29 cells treated with *Lactobacillus acidophilus* NCFM. The columns show the effect of treatment on a hypothetical gene of interest when normalising against the different reference genes based on expression levels determined in this study. The most stable gene assessed (*PGK1*) was used as the standard for the target fold changes, which were set at two-fold decrease (A), two-fold increase (B), five-fold decrease (C), and five-fold increase (D). The three lower-ranked reference genes assessed (*MVK*, *ACTB*, and *DEFB1*) are shown in order of decreasing stability as determined in this study. The control expression level was set at one as an arbitrary reference point, and is shown by the dashed red line. Error bars indicate the standard deviation.

Simulation of a two-fold regulation (up or down) of hypGOI in the HT-29 cells revealed that there was little difference in average fold change when used for normalisation of lesser-ranked reference genes (Fig. 5A, B). Due to an increase in variation in reference gene expression, statistical significance could not be achieved when normalising to the lesser-ranked genes. When simulating a five-fold decrease of hypGOI expression, statistical significance (p<0.05) could be achieved and it was not dependant on choice of reference gene used for normalisation, although the variation was noticeably greater for both *ACTB* and *DEFB1* (Fig. 5C). In the five-fold increase of hypGOI expression simulation, the large variations of *ACTB* and *DEFB1* contributed to a lack of significance in normalised gene expression values (Fig. 5D). Simulations with both five-fold decrease and increase in hypGOI expression showed relatively small differences in mean fold-change, regardless of the gene used for normalisation.

**Figure 6:**
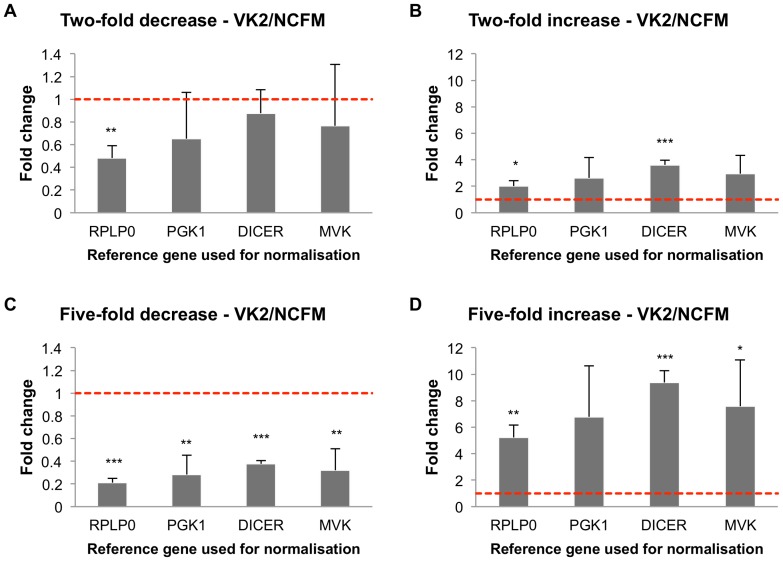
Simulation of hypGOI expression study in VK2/E6E7 cells treated with *Lactobacillus acidophilus* NCFM. The columns show the effect of treatment on a hypothetical gene of interest (hypGOI) when normalising against the different reference genes based on expression levels determined in this study. The most stable gene assessed (*RPLP0*) was used as the standard for the target fold changes, which were set at two-fold decrease (A), two-fold increase (B), five-fold decrease (C), and five-fold increase (D). The three lower-ranked reference genes assessed (*PGK1*, *DICER1*, and *MVK*) are shown in order of decreasing stability as determined in this study. The control expression level was set at one as an arbitrary reference point, and is shown by the dashed red line. Error bars indicate the standard deviation.

In the VK2/E6E7 two-fold downregulation simulation, there was no significance found after normalisation with any of the lesser-ranked reference candidates (Fig 6A). When a two-fold up regulation was simulated, significance was found only when normalised against *DICER1* (p<0.001), although the fold change was nearly double that of the intended expression levels (Fig 6B). Normalization to all lesser-ranked genes produced statistically significant results when simulating a five-fold decrease in expression, with mean fold changes comparable to intended expression levels (Fig 6C). Similarly, normalisation with the lesser-ranked genes when simulating a five-fold increase in expression gave a statistically significant upregulation, with the only exception being *PGK1*, with p = 0.06 (Fig. 6D). Normalisation to *DICER1* resulted in greatest statistical significance in this analysis; however it also showed the largest deviation from the intended expression level.

## Discussion

It has already been demonstrated that commensal bacteria are able to modulate gene expression in host cells, and real-time qPCR is currently the most powerful tool available to determine the extent of that modulation. The accuracy and reproducibility of qPCR assays depend on a number of different factors; one of the most important being a robust method of normalisation so as to minimize the impact of experimental variations. Currently the preferred normalisation involves the use of endogenous control genes, and it is now widely recognised that the choice of such a reference must be validated as to its stability in both the experimental conditions and the biological system being analysed [16]. In this study we assessed eleven different reference candidate genes for their suitability for gene expression normalisation in two epithelial cell lines that had been exposed to different probiotic and pathogenic bacteria. We found that the best reference candidates were specific to each cell line, even though both had been exposed to the same experimental conditions. This result supports findings in previous studies, and emphasises the importance of cell specific reference target validation [17], [22].

The validation included comparison of four well-known algorithms, and the results were relatively comparable across all methods. For both the colonic and vaginal cell lines the top genes (*PGK1* and *RPLP0* respectively) were identical for all four algorithms. For the colonic cell line, the bottom three ranked genes (*TMEM222, ACTB,* and *DEFB1*) were also ranked identically across all algorithms, flagging them as unlikely to be suitable as references in a qPCR study. The vaginal cell line also had the same four genes in the bottom four, but with a slightly different order (*DEFB1*, *DICER*, *MVK*, and *DROSHA*). For the remaining genes in both data sets the rankings varied. In the colonic cell line, there was a clear second and third position (*DICER* and *PPIA* respectively), with the remaining genes occupying a similar rank. In the vaginal cell line, the remaining genes could be separated into two groups, with *TMEM222*, *ACTB*, and *PPIA* holding a slightly better position than the others.

To evaluate how ranking of the reference genes influences accuracy of gene expression analysis, we performed a simulation using a hypothetical gene of interest (hypGOI). Since the fold induction of the hypGOI were generated based on PGK1 (for HT29) and RPLP0 (for VK2/E6E7) expression levels, normalisation to these genes was set as a reference point for evaluation of normalisation to the other lesser-ranked genes. The simulation revealed that the use of less stable reference genes led to less accurate quantification of expression levels of hypGOI when fold induction was two rather than five (Figs. 5 and 6). Normalisation to less stable genes can also result in decreased statistical significance for GOI expression levels when compared to the control.

The choice of whether to use one or more reference genes for analysis depends on both the power and sensitivity required for the study. Where anticipated changes in target genes are large, or when an on or off state of transcription is being measured, good normalisation can also be achieved with a well-validated single gene [13], [18]. However, when looking at target genes where the fluctuations are small, it is preferential to use a normalisation factor (NF) consisting of the geometric mean of two or more stable genes. The way the different algorithms determine which genes are the best varies, and the recommendations made in regards to how many genes are required also varies. geNorm decides on pairings based on the best correlation between the most stable genes, and then determines the combined variability to find out how many genes should be used. Although the improved algorithm can rank all candidate genes in order, the authors strongly advise against using less than two reference targets [14]. NormFinder, on the other hand, suggests pairings based on complementary intergroup variation, rather than correlation between genes. The method here is model based, and relies on the theory that normalisation with more than one reference gene is not always beneficial, and can result in transferral of error rather than correction of it [18]. Although this may hold true in most cases, this assumption may inadvertently miss true experimental error in a data set.

Although BestKeeper and comparative ΔC_q_ analyses do not provide recommendations on pairings reference candidates, they still provide valuable information on how suitable a gene would be for use in normalisation. BestKeeper also advocates the use of multiple targets, suggesting that at least three genes are needed for accurate normalisation in all cases [19]. This method provides a large amount of descriptive statistics enabling the user to determine which combination would be best by comparing results to how well they correlate the BestKeeper index. It is a dynamic system, as the results differ as combinations are changed, so it is possible to experiment with different combinations to identify the most stable overall result. Finally, the comparative ΔC_q_ method provides a simple and easy to use mathematical model that correlates well with the more complicated algorithms. Decisions can be made with regards to pairing by examining the s.d. between pairs of genes using a similar strategy by which pairings are decided using geNorm. As the weaknesses in one method can be counterbalanced by the strengths in another, we recommend that more than one method of reference gene validation should be used.

Careful selection of reference candidates for validation studies also deserves consideration. Previous studies can help indicate which genes may be suitable candidates, whereas programs such as RefGenes can provide novel suggestions based on pooled microarray data [22], [29]. In this study the latter method resulted in the new identification of *TMEM222* as the second most stable candidate for the vaginal cell line. Experiments in our laboratory also identified another novel candidate, *DICER1*, which ranked second best in the colonic cell line. And although the traditional “housekeeping genes” have received much criticism over the years, they are generally detected easily across a wide range of cell types and may be suitable for gene expression normalisation. In this study, although *ACTB* was found to be too unstable for use with colonic cells, it was determined to be one of the better options for vaginal cells. *GAPDH*, which is widely used as a reference gene in a range of studies, ranked in the middle of both data sets. Although this does not exclude this gene from use as a reference, consideration should be taken to pairing it with another reference gene, particularly where high sensitivity is required. Additionally, our results also contradict those of an earlier study, which suggested *DEFB1* as a superior reference gene to *GAPDH* in HT-29 colonic cells, as we found *GAPDH* to be better in both cell lines. Interestingly, we found *DEFB1* to have a much lower variation of expression when based on C_q_ alone (Fig. 1), although this does not appear to be a primary consideration across all algorithms tested. It should, however, be noted that the study in which this was found only compared two reference genes (*DEFB1* and *GAPDH*), and current recommendations suggest comparison of at least three [16], [29], [30]. We also found that agreement between the four algorithms increased as both more candidate genes and more experimental conditions were included in the study. This indicates robustness of a reference gene study can be improved by both the inclusion of more candidates and samples.

A further condition to consider when selecting candidates is to choose genes involved in a variety of different processes. Although we have done this in this study, we did have some candidates that are involved in similar pathways. Both *DICER1* and *DROSHA* are both involved in small RNA processing, however none of the algorithms recommended they should be used together. This is encouraging as it indicates there is unlikely to be any co-regulation between these genes affecting the overall results. This is especially relevant for the BestKeeper and geNorm analysis, as these rate combinations of reference genes on how well they correlate with each other, whereas NormFinder pairs genes according to lowest combined intergroup variation. Similarly, the combination of *GAPDH* and *PGK1*, which are both enzymes involved in glycolysis, were not recommended to be used together for the colonic cell line. In the vaginal cell line, *PGK1* and *GAPDH* were both suggested in the final combination of four genes as assessed by geNorm. However, this is based on the pairwise variation cut-off point of 0.15, which is the recommendation from geNorm for a homogenous sample set. As our data set involved the use of multiple treatments it is acceptable to increase this cut-off point. For both data sets, the combination of the best two genes only exceeded this cut-off point by very small amounts (Fig. 2C, D). Based on this, the use of fewer genes than recommended by geNorm would be acceptable for this study, and by doing so, *GAPDH* and *PGK1* would not we used concurrently as references for the same analysis.

In addition to determining the most stable gene in each data set, we also investigated the influence different species of *Lactobacillus* had on the cell's gene expression. To illustrate these differences we decided to use a radar plot, which enabled a visual comparison of how closely the results of different bacterial treatments aligned compared to the main data set (Fig. 4). By comparing how closely these lines overlapped we found that variation in reference gene stability was more affected by the cell line being analysed than the treatment performed. Variation between the cell lines is most likely due to different physiological functions, as they represented distinct anatomical locations. In addition to this, even though both cell lines are of epithelial origin, one is a columnar epithelia (colonic) and one is a squamous epithelia (vaginal). These basic differences in cell structure may also have an effect on gene expression, even when cells have similar anatomic origins [31]. Finally, gene expression may also have been affected by the differing mechanisms used to immortalise the cells. The VK2/E6E7 cell line was immortalised by transformation with the human papilloma virus oncogenes E6 and E7. This process deregulates cell cycle control by deactivation of key tumour suppressor proteins such as p53 and the retinoblastoma tumour suppressor protein, among others, and has the potential to enable a range of mutations that could disrupt gene regulation [32], [33]. In contrast, the HT-29 cell line originates from colorectal adenocarcinoma cells that are hypertriploid, with a range of chromosomal abnormalities that may also alter gene regulation patterns. Although it has been demonstrated that expression patterns of carcinoma cells correlates most strongly to its tissue of origin, it has also been shown that there are clear differences in gene expression patterns of carcinoma cell lines both in relation to their tissue of origin and in relation to each other [34]–[36]. Better understanding of these expression patterns in relation to reference gene expression may be important for future gene expression studies. Reference genes validated for a particular cell line could be used for a range of different experiments without a full validation if a primary screen supports previously determined stability. Additional studies, including investigation of a greater range of both treatments and cell lines, would be required to confirm this.

In conclusion, suitable reference candidates were selected evident for use in colonic and vaginal cells after treatment with *L. rhamnosus*, *L. acidophilus*, and heat-killed *E. coli*. *PGK1* was the most stably expressed in HT-29 cells, and *RPLP0* was the best choice for the VK2/E6E7 cells. Additionally, two novel genes (*DICER1* for HT-29 cells and *TMEM222* for VK2/E6E7 cells) were identified as good choices as references. Additional high-ranking reference genes should be used where greater sensitivity is required. Our results suggest that that the top ranked gene should be used for normalisation, along with any other of the genes with a geometric rank of less than five. The HT-39 data set consists of *PGK1* paired with *DICER1*, *PPIA*, *RPLP0*, or *POLR2A*, and the VK2/E6E7 data set, *RPLP0* should be used, combined with *TMEM222*, *ACTB*, *PPIA*, *GAPDH*, or *PGK1* as required. Our studies also indicate that the cell type being investigated is more important than the treatment itself when determining a suitable reference for qPCR studies. Use of these reference candidates with other cell lines undergoing similar treatments should not be considered unless an independent validation was conducted.

## Supporting Information

S1 Fig **Division of experiments for secondary analysis.** Green circles indicate experiments that were only included in the *Lactobacillus acidophilus* NCFM secondary analysis, and blue circles indicate experiments that were only used in the *L. rhamnosus* GR-1 secondary analysis. Orange circles indicate experiments that were used in both secondary analyses. GR-12: Heat-killed *Escherichia coli* GR-12.
(TIF)

S2 Fig **geNorm and NormFinder analyses for the HT-29 cells**. geNorm M analysis is shown in order of increasing stability for the NCFM (A) and GR-1 (B) subgroups. Pairwise variation analysis is summarised for the NCFM (C) and GR-1 (D) subgroups, with the geNorm acceptable variation cut-off of 0.15 shown by the dashed red line. A summary of the NormFinder stabilities is shown for the NCFM (E) and GR-1 (F) subgroups, with the best ranking candidate genes indicated in red.
(TIF)

S3 Fig **geNorm and NormFinder analyses for the VK2/E6E7 cells**. geNorm M analysis is shown in order of increasing stability for the NCFM (A) and GR-1 (B) subgroups. Pairwise variation analysis is summarised for the NCFM (C) and GR-1 (D) subgroups, with the geNorm acceptable variation cut-off of 0.15 shown by the dashed red line. A summary of the NormFinder stabilities is shown for the NCFM (E) and GR-1 (F) subgroups, with the best ranking candidate genes indicated in red.
(TIF)

S1 Table **Summary of key statistics after BestKeeper analysis – HT29 data set.** The standard deviation (s.d.) corresponds to the individual gene stability and the coefficient of correlation, with its respective p-value, corresponds to how closely the candidate reference gene resembles the BestKeeper ideal normalisation factor after repeated pair-wise analysis. The power value is determined by regression analysis, using fold change (x-fold) as a reference point, and a smaller value indicates a better reference candidate. Values for genes eliminated in earlier stages of analysis are not shown.
(DOCX)

S2 Table **Mean standard deviation (s.d.) of reference genes using the comparative ΔC_q_ method – HT29 data set.**
(DOCX)

S3 Table **Comprehensive ranking of reference gene candidates by calculation of a geometric mean – HT29 data set.**
(DOCX)

S4 Table **Summary of key statistics after BestKeeper analysis – VK2/E6E7 data set.** The standard deviation (s.d.) corresponds to the individual gene stability and the coefficient of correlation, with its respective p-value, corresponds to how closely the candidate reference gene resembles the BestKeeper ideal normalisation factor after repeated pair-wise analysis. The power value is determined by regression analysis, using fold change (x-fold) as a reference point, and a smaller value indicates a better reference candidate. Values for genes eliminated in earlier stages of analysis are not shown.
(DOCX)

S5 Table **Mean standard deviation (s.d.) of reference genes using the comparative ΔC_q_ method – VK2/E6E7 data set.**
(DOCX)

S6 Table **Comprehensive ranking of reference gene candidates by calculation of a geometric mean – VK2/E6E7 data set.**
(DOCX)

S1 File **Supplementary information.**
(DOCX)
