# Supplementary figures and images for: Reference Gene Selection for qPCR Is Dependent on Cell Type Rather than Treatment in Colonic and Vaginal Human Epithelial Cell Lines
Source: PLoS One. 2014 Dec 19;9(12):e115592. doi: 10.1371/journal.pone.0115592 (PMC4272277; doi:10.1371/journal.pone.0115592)

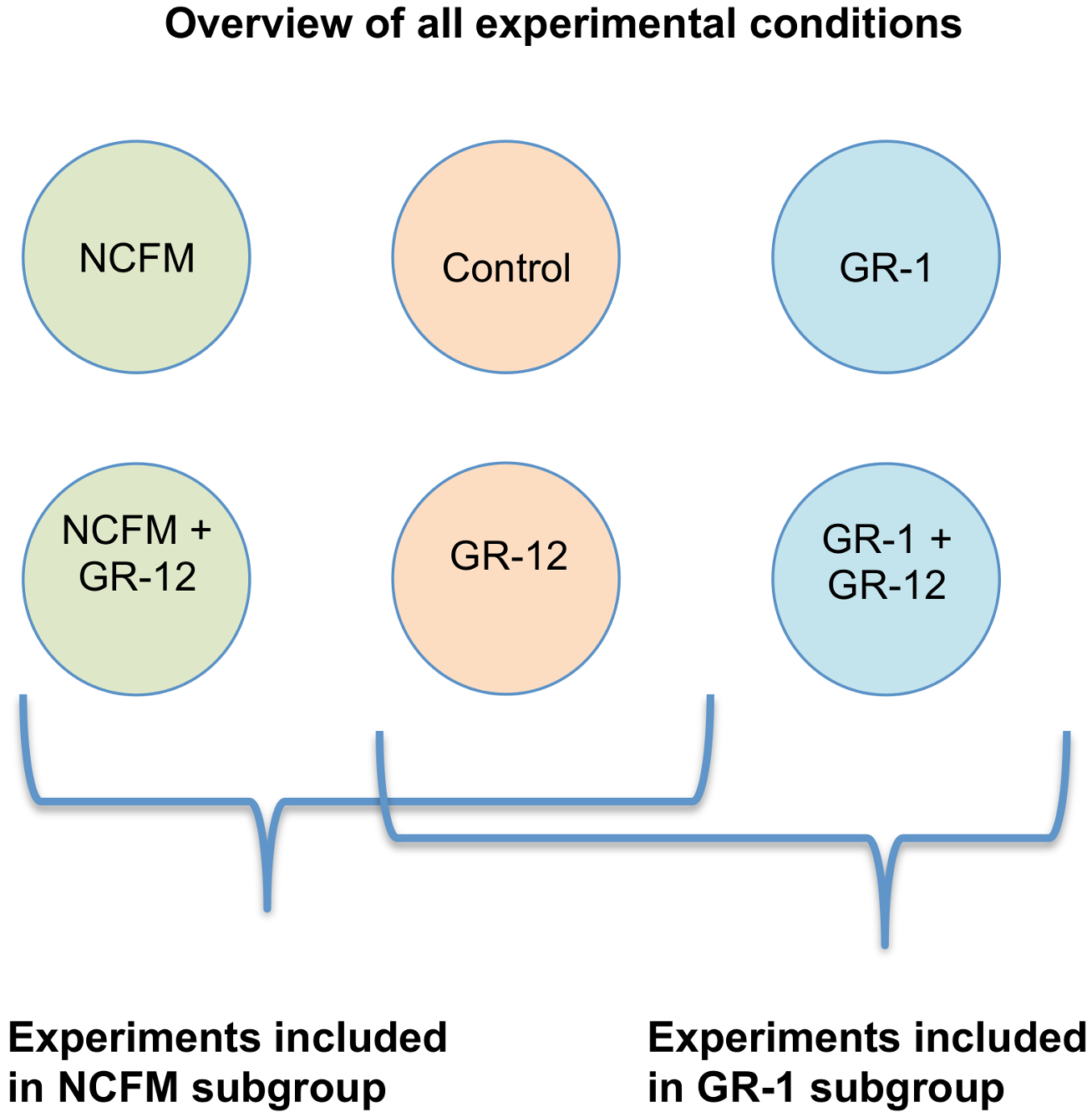

Supplement: S1 Fig — Division of experiments for secondary analysis. Green circles indicate experiments that were only included in the Lactobacillus acidophilus NCFM secondary analysis, and blue circles indicate experiments that were only used in the L. rhamnosus GR-1 secondary analysis. Orange circles indicate experiments that were used in both secondary analyses. GR-12: Heat-killed Escherichia coli GR-12. (TIF) [file pone.0115592.s001.tif]

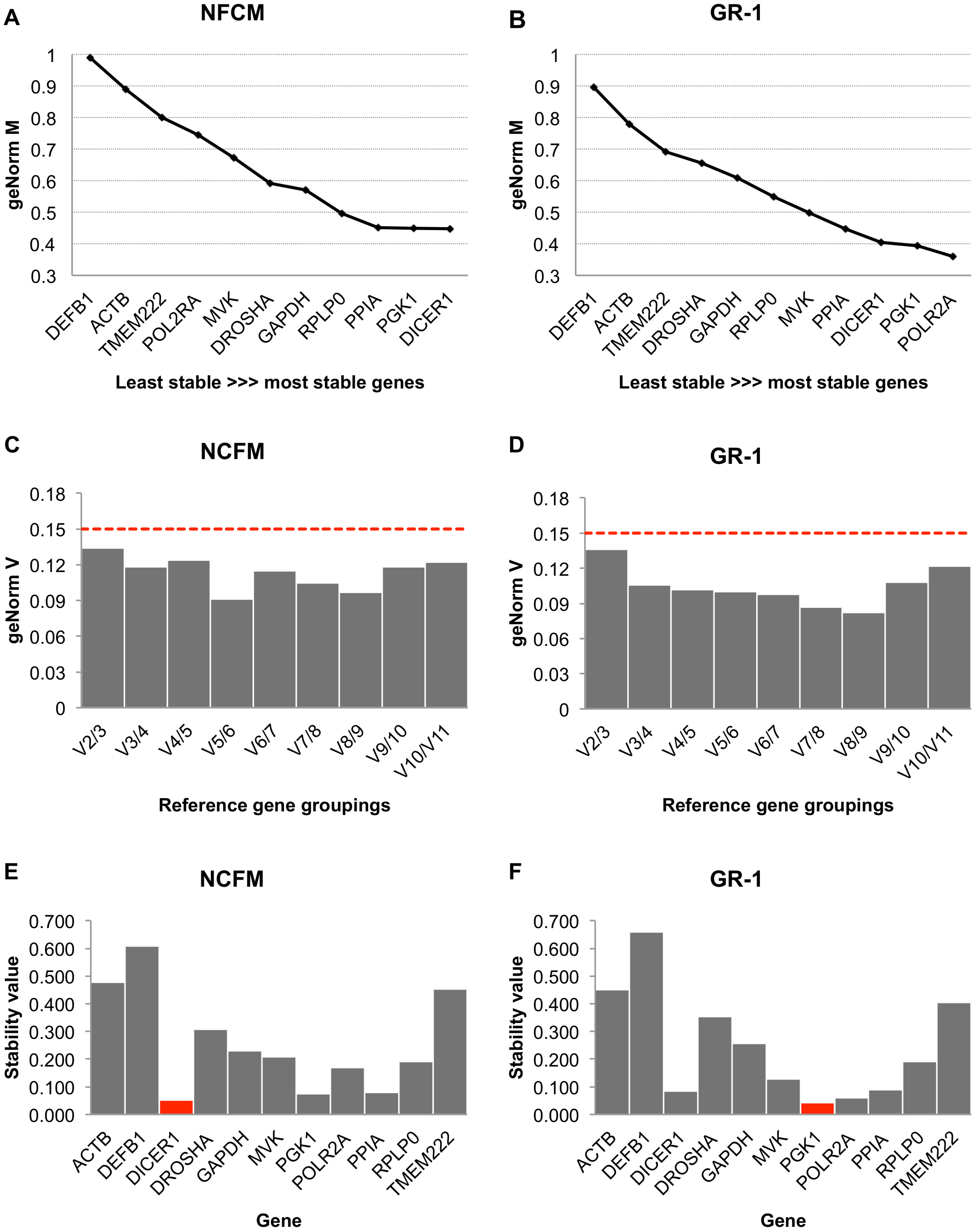

Supplement: S2 Fig — geNorm and NormFinder analyses for the HT-29 cells. geNorm M analysis is shown in order of increasing stability for the NCFM (A) and GR-1 (B) subgroups. Pairwise variation analysis is summarised for the NCFM (C) and GR-1 (D) subgroups, with the geNorm acceptable variation cut-off of 0.15 shown by the dashed red line. A summary of the NormFinder stabilities is shown for the NCFM (E) and GR-1 (F) subgroups, with the best ranking candidate genes indicated in red. (TIF) [file pone.0115592.s002.tif]

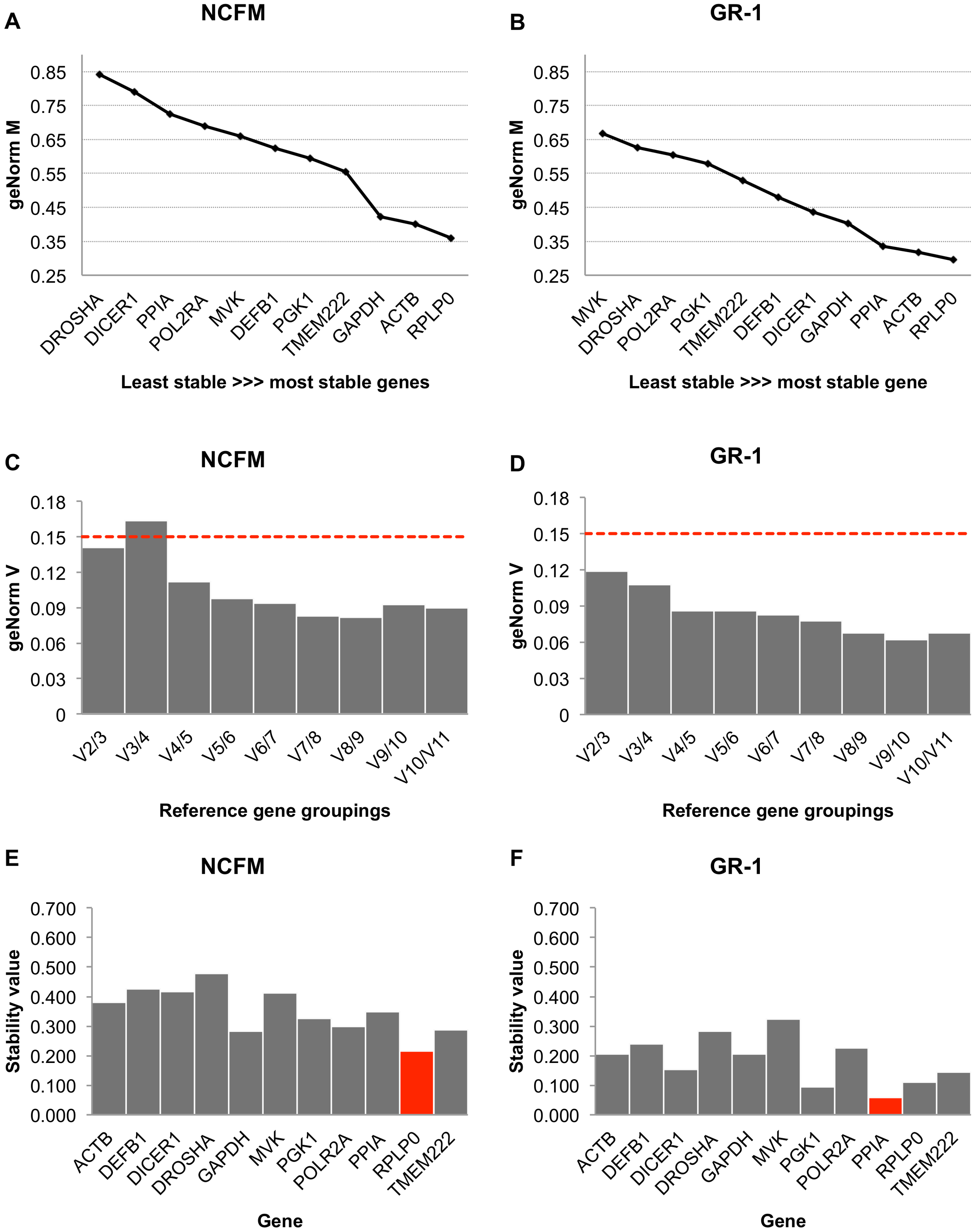

Supplement: S3 Fig — geNorm and NormFinder analyses for the VK2/E6E7 cells. geNorm M analysis is shown in order of increasing stability for the NCFM (A) and GR-1 (B) subgroups. Pairwise variation analysis is summarised for the NCFM (C) and GR-1 (D) subgroups, with the geNorm acceptable variation cut-off of 0.15 shown by the dashed red line. A summary of the NormFinder stabilities is shown for the NCFM (E) and GR-1 (F) subgroups, with the best ranking candidate genes indicated in red. (TIF) [file pone.0115592.s003.tif]
