# Supplementary material for: Reference Gene Selection for qPCR Is Dependent on Cell Type Rather than Treatment in Colonic and Vaginal Human Epithelial Cell Lines
Source: PLoS One. 2014 Dec 19;9(12):e115592. doi: 10.1371/journal.pone.0115592 (PMC4272277; doi:10.1371/journal.pone.0115592)
Supplement: S1 Table — Summary of key statistics after BestKeeper analysis – HT29 data set. The standard deviation (s.d.) corresponds to the individual gene stability and the coefficient of correlation, with its respective p-value, corresponds to how closely the candidate reference gene resembles the BestKeeper ideal normalisation factor after repeated pair-wise analysis. The power value is determined by regression analysis, using fold change (x-fold) as a reference point, and a smaller value indicates a better reference candidate. Values for genes eliminated in earlier stages of analysis are not shown. (DOCX) [file pone.0115592.s004.docx]

| **NCFM** | | | | | | |
| --- | --- | --- | --- | --- | --- | --- |
|  | **DICER1** | **MVK** | **PGK1** | **POLR2A** | **PPIA** | **RPLP0** |
| s.d. [± Cq] | 0.69 | 0.75 | 0.96 | 1.03 | 0.73 | 0.90 |
| coeff. of corr. [r] | 0.977 | 0.817 | 0.987 | 0.920 | 0.957 | 0.864 |
| p-value | 0.001 | 0.001 | 0.001 | 0.001 | 0.001 | 0.001 |
| Power [x-fold] | 1.78 | 1.77 | 2.19 | 2.37 | 1.77 | 1.93 |
| **GR-1** | | | | | | |
|  | **DICER1** | **MVK** | **PGK1** | **POLR2A** | **PPIA** | **RPLP0** |
| s.d. [± Cq] | 0.58 | 0.61 | 0.80 | 0.76 | 0.54 | 0.85 |
| coeff. of corr. [r] | 0.917 | 0.776 | 0.984 | 0.959 | 0.905 | 0.886 |
| p-value | 0.001 | 0.003 | 0.001 | 0.001 | 0.001 | 0.001 |
| Power [x-fold] | 1.75 | 1.80 | 2.23 | 2.09 | 1.65 | 2.13 |
