# Supplementary material for: Reference Gene Selection for qPCR Is Dependent on Cell Type Rather than Treatment in Colonic and Vaginal Human Epithelial Cell Lines
Source: PLoS One. 2014 Dec 19;9(12):e115592. doi: 10.1371/journal.pone.0115592 (PMC4272277; doi:10.1371/journal.pone.0115592)
Supplement: S2 Table — Mean standard deviation (s.d.) of reference genes using the comparative ΔCq method – HT29 data set. (DOCX) [file pone.0115592.s005.docx]

| **NCFM** | | **GR-1** | |
| --- | --- | --- | --- |
| **Gene** | **Mean s.d.** | **Gene** | **Mean s.d.** |
| PGK1 | 0.717 | PGK1 | 0.686 |
| DICER1 | 0.721 | POLR2A | 0.689 |
| PPIA | 0.739 | DICER1 | 0.713 |
| GAPDH | 0.824 | PPIA | 0.747 |
| DROSHA | 0.882 | GAPDH | 0.801 |
| POLR2A | 0.888 | RPLP0 | 0.826 |
| RPLP0 | 0.903 | MVK | 0.863 |
| MVK | 0.914 | DROSHA | 0.934 |
| TMEM222 | 1.181 | TMEM222 | 1.049 |
| ACTB | 1.230 | ACTB | 1.202 |
| DEFB1 | 1.424 | DEFB1 | 1.500 |
