# Supplementary material for: Reference Gene Selection for qPCR Is Dependent on Cell Type Rather than Treatment in Colonic and Vaginal Human Epithelial Cell Lines
Source: PLoS One. 2014 Dec 19;9(12):e115592. doi: 10.1371/journal.pone.0115592 (PMC4272277; doi:10.1371/journal.pone.0115592)
Supplement: S3 Table — Comprehensive ranking of reference gene candidates by calculation of a geometric mean – HT29 data set. (DOCX) [file pone.0115592.s006.docx]

| **NCFM** | | | | | |
| --- | --- | --- | --- | --- | --- |
| **geNorm** | **BestKeeper** | **NormFinder** | **ΔC_q_** | **Ranking** | **Mean** |
| DICER1 | PGK1 | DICER1 | PGK1 | **PGK1** | 1.414 |
| PGK1 | DICER1 | PGK1 | DICER1 | **DICER1** | 1.414 |
| PPIA | PPIA | PPIA | PPIA | **PPIA** | 3.000 |
| RPLP0 | RPLP0 | POLR2A | GAPDH | **RPLP0** | 4.864 |
| GAPDH | MVK | RPLP0 | DROSHA | **GAPDH** | 5.595 |
| DROSHA | POLR2A | MVK | POLR2A | **POLR2A** | 5.826 |
| MVK | GAPDH | GAPDH | RPLP0 | **MVK** | 6.402 |
| POLR2A | DROSHA | DROSHA | MVK | **DROSHA** | 6.620 |
| TMEM222 | TMEM222 | TMEM222 | TMEM222 | **TMEM222** | 9.000 |
| ACTB | ACTB | ACTB | ACTB | **ACTB** | 10.00 |
| DEFB1 | DEFB1 | DEFB1 | DEFB1 | **DEFB1** | 11.00 |
| **GR-1** | | | | | |
| **geNorm** | **BestKeeper** | **NormFinder** | **ΔC_q_** | **Ranking** | **Mean** |
| POLR2A | RPLP0 | PGK1 | PGK1 | **PGK1** | 1.414 |
| PGK1 | PGK1 | POLR2A | POLR2A | **POLR2A** | 2.000 |
| DICER | PPIA | DICER1 | DICER1 | **DICER1** | 3.409 |
| PPIA | POLR2A | PPIA | PPIA | **PPIA** | 3.722 |
| MVK | DICER1 | MVK | GAPDH | **RPLP0** | 3.834 |
| RPLP0 | MVK | RPLP0 | RPLP0 | **MVK** | 5.692 |
| GAPDH | GAPDH | GAPDH | MVK | **GAPDH** | 6.435 |
| DROSHA | DROSHA | DROSHA | DROSHA | **DROSHA** | 8.000 |
| TMEM222 | TMEM222 | TMEM222 | TMEM222 | **TMEM222** | 9.000 |
| ACTB | ACTB | ACTB | ACTB | **ACTB** | 10.00 |
| DEFB1 | DEFB1 | DEFB1 | DEFB1 | **DEFB1** | 11.00 |
