# Supplementary material for: Reference Gene Selection for qPCR Is Dependent on Cell Type Rather than Treatment in Colonic and Vaginal Human Epithelial Cell Lines
Source: PLoS One. 2014 Dec 19;9(12):e115592. doi: 10.1371/journal.pone.0115592 (PMC4272277; doi:10.1371/journal.pone.0115592)
Supplement: S4 Table — Summary of key statistics after BestKeeper analysis – VK2/E6E7 data set. The standard deviation (s.d.) corresponds to the individual gene stability and the coefficient of correlation, with its respective p-value, corresponds to how closely the candidate reference gene resembles the BestKeeper ideal normalisation factor after repeated pair-wise analysis. The power value is determined by regression analysis, using fold change (x-fold) as a reference point, and a smaller value indicates a better reference candidate. Values for genes eliminated in earlier stages of analysis are not shown. (DOCX) [file pone.0115592.s007.docx]

| **NCFM** | | | | | | |
| --- | --- | --- | --- | --- | --- | --- |
|  | **ACTB** | **DEFB1** | **PGK1** | **PPIA** | **RPLP0** | **TMEM222** |
| s.d. [± Cq] | 0.71 | 0.47 | 0.94 | 0.95 | 0.82 | 0.83 |
| coeff. of corr. [r] | 0.926 | 0.864 | 0.934 | 0.852 | 0.986 | 0.889 |
| p-value | 0.001 | 0.001 | 0.001 | 0.001 | 0.001 | 0.001 |
| Power [x-fold] | 1.82 | 1.43 | 2.35 | 2.14 | 2.04 | 1.91 |
| **GR-1** | | | | | | |
|  | **ACTB** | **DEFB1** | **DICER1** | **GAPDH** | **PPIA** | **RPLP0** |
| s.d. [± Cq] | 0.74 | 0.44 | 0.91 | 0.92 | 0.70 | 0.73 |
| coeff. of corr. [r] | 0.965 | 0.906 | 0.945 | 0.964 | 0.975 | 0.975 |
| p-value | 0.001 | 0.003 | 0.001 | 0.001 | 0.001 | 0.001 |
| Power [x-fold] | 1.90 | 1.45 | 2.19 | 2.19 | 1.90 | 1.96 |
