# Supplementary material for: Reference Gene Selection for qPCR Is Dependent on Cell Type Rather than Treatment in Colonic and Vaginal Human Epithelial Cell Lines
Source: PLoS One. 2014 Dec 19;9(12):e115592. doi: 10.1371/journal.pone.0115592 (PMC4272277; doi:10.1371/journal.pone.0115592)
Supplement: S5 Table — Mean standard deviation (s.d.) of reference genes using the comparative ΔCq method – VK2/E6E7 data set. (DOCX) [file pone.0115592.s008.docx]

| **NCFM** | | **GR-1** | |
| --- | --- | --- | --- |
| **Gene** | **Mean s.d.** | **Gene** | **Mean s.d.** |
| RPLP0 | 0.669 | PPIA | 0.514 |
| TMEM222 | 0.720 | RPLP0 | 0.565 |
| GAPDH | 0.766 | DICER1 | 0.603 |
| POL2R2A | 0.790 | TMEM222 | 0.619 |
| PGK | 0.798 | ACTB | 0.637 |
| PPIA | 0.810 | GAPDH | 0.679 |
| ACTB | 0.823 | PGK | 0.686 |
| MVK | 0.899 | POLR2A | 0.695 |
| DEFB1 | 0.930 | DEFB1 | 0.698 |
| DICER1 | 0.960 | DROSHA | 0.765 |
| DROSHA | 1.079 | MVK | 0.838 |
