# Supplementary material for: Reference Gene Selection for qPCR Is Dependent on Cell Type Rather than Treatment in Colonic and Vaginal Human Epithelial Cell Lines
Source: PLoS One. 2014 Dec 19;9(12):e115592. doi: 10.1371/journal.pone.0115592 (PMC4272277; doi:10.1371/journal.pone.0115592)
Supplement: S6 Table — Comprehensive ranking of reference gene candidates by calculation of a geometric mean – VK2/E6E7 data set. (DOCX) [file pone.0115592.s009.docx]

| **NCFM** | | | | | |
| --- | --- | --- | --- | --- | --- |
| **geNorm** | **BestKeeper** | **NormFinder** | **ΔC_q_** | **Ranking** | **Mean** |
| RPLP0 | RPLP0 | RPLP0 | RPLP0 | **RPLP0** | 1.000 |
| ACTB | ACTB | GAPDH | TMEM222 | **TMEM222** | 3.310 |
| GAPDH | PGK1 | TMEM222 | GAPDH | **GAPDH** | 3.464 |
| TMEM222 | DEFB1 | POL2RA | POLR2A | **ACTB** | 3.742 |
| PGK1 | TMEM222 | PGK1 | PGK1 | **PGK1** | 4.401 |
| DEFB1 | PPIA | PPIA | PPIA | **POLR2A** | 5.826 |
| MVK | MVK | ACTB | ACTB | **PPIA** | 6.640 |
| POLR2A | GAPDH | MVK | MVK | **DEFB1** | 6.817 |
| PPIA | POLR2A | DICER1 | DEFB1 | **MVK** | 7.483 |
| DICER1 | DICER1 | DEFB1 | DICER1 | **DICER1** | 9.740 |
| DROSHA | DROSHA | DROSHA | DROSHA | **DROSHA** | 11.00 |
| **GR-1** | | | | | |
| **geNorm** | **BestKeeper** | **NormFinder** | **ΔC_q_** | **Ranking** | **Mean** |
| RPLP0 | RPLP0 | PPIA | PPIA | **RPLP0** | 1.565 |
| ACTB | ACTB | PGK1 | RPLP0 | **PPIA** | 1.732 |
| PPIA | PPIA | RPLP0 | DICER1 | **ACTB** | 3.440 |
| GAPDH | GAPDH | TMEM222 | TMEM222 | **DICER** | 4.401 |
| DICER1 | DICER1 | DICER1 | ACTB | **GAPDH** | 4.899 |
| DEFB1 | DEFB1 | GAPDH | GAPDH | **PGK1** | 5.292 |
| TMEM222 | PGK1 | ACTB | PGK1 | **TMEM222** | 5.635 |
| PGK1 | POLR2A | POLR2A | POLR2A | **DEFB1** | 7.348 |
| POLR2A | TMEM222 | DEFB1 | DEFB1 | **POLR2A** | 8.239 |
| DROSHA | MVK | DROSHA | DROSHA | **DROSHA** | 10.24 |
| MVK | DROSHA | MVK | MVK | **MVK** | 10.74 |
