# Supplementary material for: Reference Gene Selection for qPCR Is Dependent on Cell Type Rather than Treatment in Colonic and Vaginal Human Epithelial Cell Lines
Source: PLoS One. 2014 Dec 19;9(12):e115592. doi: 10.1371/journal.pone.0115592 (PMC4272277; doi:10.1371/journal.pone.0115592)
Supplement: S1 File — Supplementary information. (DOCX) [file pone.0115592.s010.docx]

**Supplementary Information**

1. **Division of experiments for subgroup analysis**

To try and determine the impact of different bacterial treatments on our analyses, we divided our qPCR data set into two groups. Our original data set involved six total treatment groups with three replicates (n=18), and this was split into two groups of four treatment groups with three replicates (n=12) based on the *Lactobacillus* species used in treatment (Figure S1). As the premise of a reference gene analysis assumes no change, each sample is assumed to be equivalent, regardless of treatment. The subgroups were designated NCFM, for *Lactobacillus acidophilus* NCFM, and GR-1, for *Lactobacillus rhamnosus* GR-1. After division, each subgroup was analysed using the four analysis methods described in the main report, and a geometric mean for each gene was determined.

1. **Colonic HT-29 cell line subgroup analyses**

*geNorm analysis*

Both the subgroups in the HT-29 data set showed increased stability in geNorm analysis as when compared to the main data set, with both subgroups rating under the 0.15 geNorm V cutoff point using only two genes (Figure S2A-D; Figure 2A and C). The NCFM rankings were extremely similar to the complete data set, with only a slight reordering in the top four genes. In the GR-1 data set, there were more changes, with the most significant being movement of *POLR2A* from 8^th^ to 1^st^. Aside from this, no other gene moved more than two ranking places, and the lowest three genes were identical across all data sets.

*NormFinder analysis*

NormFinder analysis between all data sets analysed showed very similar results (Figure S2E-F; Figure 3A). Like the geNorm analysis, most differences seen were in the best ranking genes, although here positions did not change by more than two ranking places in any of the data sets. *POLR2A* ranked best in the GR-1 data set, going from 4^th^ place to 2^nd^ place. *DICER1* ranked better than *PGK1* in the NCFM data set.

*BestKeeper analysis*

BestKeeper analysis followed a similar pattern to the other two analyses, with relatively small changes among the better ranking genes. The same genes occupied the top six rankings, but in slightly different spots (Table S1; Table 3). Most notably, *RPLP0* ranked best in the Gr-1 data set, up from 4^th^ in the NCFM data set and 5^th^ in the overall data set. Stability overall was better in the Gr-1 data set, with the s.d. for each gene being lower.

*ΔC_q_ analysis*

This analysis also indicated increased stability in the GR-1 data set, with lower mean s.d. values in this data set overall. *POLR2A* again ranked much higher in the GR-1 data set, going to second place, compared to 6^th^ in the NCFM data set, and 5^th^ overall. *DROSHA* ranked better in the NCFM data set, coming in 5^th^ position, as compared to 8^th^ in both the other data sets (Table S2; Table 4).

*HT29 summary*

Although there were some differences in the individual rankings, the overall analysis gave the same genes in the bottom three places in both the overall analysis and the subgroup analyses. The same gene (*PGK1*) occupied top place in all analyses, although it shared this with *DICER1* in the NCFM subset. Other genes showed slight variations in placement depending on the subgroup, with the most notable being *POLR2A*, which ranked much better in the GR-1 subgroup than in other analyses (Table S3; Table 5).

**3. Vaginal VK2/E6E7 cell line subgroup analyses**

*geNorm analysis*

The NCFM subgroup had a very similar pattern to the full analysis, both in the overall ranking of genes and the pattern of stability (Figure S3; Figure 2). There were mostly minor changes to the ranking, with genes that ranked poorly in the full analysis also ranking poorly in the subgroup analysis, and vice versa. Interestingly, geNorm evaluation determined the use of two reference genes gave less variability than the use of three reference genes in this subgroup. The GR-1 subgroup showed greater overall stability than both the NCFM subgroup and the full analysis, with significantly lower geNorm M and V values. There were also more changes to the overall rankings in this subgroup, although the top two genes (*RPLP0* and *ACTB*) remained the same across all three analyses. One of the biggest ranking changes was *PPIA*, which ranked poorly in the NCFM group (9^th^) and well in the GR-1 group (3^rd^), and this ranked middling (6^th^) in the overall analysis. Other changes include to rankings of *MVK* and *DICER1*, which ranged from poor to middling, depending on the analysis group. Other ranking changes were relatively minor.

*NormFinder analysis*

Like the geNorm analysis, the NormFinder analysis of the NCFM subgroup was very similar to the full analysis, with the only major change being movement of *GAPDH* from 6^th^ to 2^nd^ (Figure S3; Figure 3). Also similar to geNorm analysis, the GR-1 subgroup showed much greater overall stability, having significantly lower stability values. Also, there were more positional changes, with *PPIA* and *PGK1* both ranking as more stable than RPLP0 in this analysis. However, it should be noted that *RPLP0* still had greater overall stability than both the NCFM subgroup and the full analysis despite the drop in ranking. Most other changes were not of major consequence, with changes in ranking between good to middling, or middling to poor.

*BestKeeper analysis*

In the BestKeeper analysis, *RPLP0* and *ACTB* were found to be the most stable candidates regardless of which group was analysed (Table S4; Table 3). Similarly, *DROSHA* was clearly the poorest choice. *DEFB1* and *TMEM222* moved into the top six in the NCFM subgroup, displacing *GAPDH* and *POLR2A*. In the GR-1 subgroup *DEFB1* improved position along with *DICER1*, with *PGK1* and *POLR2A* dropping down the ranks.

*ΔC_q_ analysis*

Similar to the other analyses, the GR-1 subgroup showed overall greater stability with lower mean standard deviations (Table S5; Table 4). The NCFM subgroup again showed greatest similarity to the overall analysis, with the same two genes in the top two positions (*RPLP0* and *TMEM222*), and the same four genes ranked last, albeit with minor positional changes. In the middle rankings, changes were relatively minor. The analysis of the GR-1 subgroup placed *PPIA* slightly ahead of *RPLP0*. *DICER1* also made a big jump in the rankings, improving to place 3^rd^ from ranking very low in both the NCFM subgroup (10^th^) and the overall analysis (9^th^). Other genes showed relatively small positional changes.

*VK2/E6E7 summary*

All analyses ranked RPLP0 as the most stable reference gene candidate after geometric ranking, although in the GR-1 subgroup this was closely followed by PPIA, which ranked 4^th^ overall, and 7^th^ in the NCFM subgroup (Table S6). DROSHA, MVK and DEFB1 all ranked in the bottom four for all three analyses, although with slightly different rankings. DICER1 also showed a large overall ranking change, placing 4^th^ in the GR-1 subgroup, 10^th^ in the NCFM subgroup, and 9^th^ overall. Other genes showed slight positional changes, with very little difference in actual ranking points.
